# Impact of Semiochemicals Binding to Fel d 1 on Its 3D Conformation and Predicted B-Cell Epitopes Using Computational Approaches

**DOI:** 10.3390/ijms241411685

**Published:** 2023-07-20

**Authors:** Rajesh Durairaj, Patrick Pageat, Cécile Bienboire-Frosini

**Affiliations:** 1 Department of Bioinformatics and Chemical Communication (D-BICC), Research Institute in Semiochemistry and Applied Ethology (IRSEA), Quartier Salignan, 84400 Apt, France; 2 Research and Education Board, Research Institute in Semiochemistry and Applied Ethology (IRSEA), Quartier Salignan, 84400 Apt, France; p.pageat@group-irsea.com; 3 Department of Molecular Biology and Chemical Communication (D-BMCC), Research Institute in Semiochemistry and Applied Ethology (IRSEA), Quartier Salignan, 84400 Apt, France; c.frosini@group-irsea.com

**Keywords:** Fel d 1, major cat allergen, conformational epitopes, molecular dynamic simulation, IgE, semiochemicals

## Abstract

The major cat allergen Fel d 1 is a tetrameric glycoprotein from the secretoglobin superfamily. Fel d 1’s biological role is unknown, but it has been previously shown that it participates in semiochemical binding/transportation. Fel d 1 has linear epitopes, but its conformational epitope sites remain unclear. In this study, we predicted the B-cell epitopes of Fel d 1 and explored semiochemical dynamics with epitopes using bioinformatics tools. The epitope residues were tabulated for chains 1 and 2 and the heterodimers of Fel d 1. The residual interactions of Fel d 1 with IgE were evaluated, and the prominent epitope sites were predicted. The molecular dynamics simulation (MDS) of Fel d 1 was performed with seven reported semiochemicals to evaluate the Fel d 1–ligand complex stability and decipher the semiochemical effect on Fel d 1 conformational epitopes. Fel d 1–lauric acid, Fel d 1–oleic acid, and Fel d 1–progesterone showed more stability and less fluctuation than other compounds. Fel d 1–linoleic acid and Fel d 1–pregnenolone displayed the most unstable complex with fluctuations. The effects of conformational changes on epitopes are discussed. All the ligand complexes drive substantial fluctuation towards the functionally exposed IgE-binding epitopes. Fel d 1 could be examined for its ligand-binding and conformational changes caused by mutations of B-cell epitopes.

## 1. Introduction

Domestic cats (*Felis catus*) secrete and release abundant quantities of the major cat allergen (Fel d 1) in the dust and air [1]. Cats are the prime source of indoor respiratory allergens after house mites and raise major health problems for humans [2]. Fel d 1 is an important cause of sensitization and allergic diseases such as asthma, allergic rhinitis, and conjunctivitis [3,4]. As reported in 2006 [5,6], the prevalence of sensitization to cat allergens has increased to 10–30% in western countries.

Several animal allergens belong to the lipocalin superfamily [7], but Fel d 1 is a tetrameric glycoprotein of the secretoglobin superfamily [4]. The allergen Fel d 1 is about 35–38 kDa and consists of two identical heterodimers (subunits A and B) with the dimerization interface [8,9]. Each heterodimer consists of two polypeptide chains linked by three disulfide bridges: chain 1 contains 70 residues; chain 2 contains 90 or 92 residues; and the two chains are encoded by independent genes [10,11]. Truncated Fel d 1 dimer forms have also been identified in natural feline samples (particularly anal sacs) and house dust [12,13].

Fel d 1 is produced by the lacrimal glands, salivary glands, and sebaceous glands. It is secreted from the skin and is located on the fur [1,14,15,16]. Previous studies have confirmed that the Fel d 1 concentration on cats’ fur is higher in males than in females [17,18]. This was contrary to the report by Kelly et al. regarding cat-to-cat variability of Fel d 1 levels, which observed that they are increased in the fur of domestic cats but are unrelated to the breed, sex, or age, and that the hormonal status can affect urinary Fel d 1 levels in male cats [19]. The secretion of Fel d 1 depends on androgen control [20]. The biological function of Fel d 1 is still unclear, but the protein is produced in the same areas known to release cat semiochemicals, including the facial area, the podial complex, and the perianal zone, which contain glands that secrete chemical cues involved in cat intraspecific communications [16,21]. In addition, our previous studies suggested that Fel d 1 plays a role in cats’ chemical communication via semiochemical transport/processing [22,23] and that the structural similarity between Fel d 1 and ABP also supports its function in intraspecific communications [22]. Fel d 1 immunological features have also been linked to cat sex and behaviour [24].

Fel d 1 is well known for its allergenic potential and elicits the production of specific immunoglobulin E (IgE) in 85–95% of allergic patients [25]. It is essential to predict the B-cell epitopes for immunotherapy advances and immunoglobulin deactivation/inhibition [26]. The Fel d 1 conformational epitope site is significantly important to the study of the nature of residues and their localization in the protein. In general, B-cell epitopes can be contiguous/continuous (a linear stretch of amino acid residues linked by a peptide bond) or non-contiguous/discontinuous (several spatially separate residues combined through conformational folding of the polypeptide chain) [27]. The B-cell epitopes are recognized and discriminated by immunoglobins. It is hard to find the discontinuous amino acid residues and their interaction with IgE-specific monoclonal antibodies (mAb). The linear epitope in silico predictions (hydrophilicity, secondary structure, and surface-exposed residues) are like T-cell epitope predictions, but the B-cell conformational epitopes are predicted based on the three-dimensional (3D) structure of a protein [26,28]. The specificity of the conformational epitope sites depends on the spatial folding and the conformation of the contributing individual sequential epitopes [27]. The conformational epitope sites are predicted based on the 3D structure of the protein to determine the surface accessibility and the residual propensity, the spatial proximity, and the contacts with the protein. Several studies have identified the T-cell reactive peptides/epitopes of rFel d 1, and their IgE binding activities have been described [29,30,31,32,33,34]. Other studies have reported IgE binding B-cell epitope sites and epitope mapping in Fel d 1 [8,35,36,37]. The conformational epitope sites are present in chain 1 and not mentioned in chain 2. B-cell epitopes have also been studied using mouse monoclonal antibodies developed by Chapman et al. [38]. Among these six monoclonal antibodies (6F9, 3E4, 1G9, 8F3, 2H4, and 10G7), Slunt et al. [39] showed that the first three bind to recombinant chain 1 and none to recombinant chain 2, proving the importance of chain 1 as a site of localization of B-cell epitopes on Fel d 1. The other three monoclonal Abs, therefore, appeared to be conformational. Later, Batard et al. [40] demonstrated the existence of a partially cryptic epitope in Fel d 1 which depended on the structural features of the molecule. 

In addition, our previous report suggested that Fel d 1 showed very good binding affinity and molecular interactions with a few fatty acids and steroids, notably some that are involved in feline chemical communication [23]. Other mammalian respiratory allergens have also been reported as being capable of binding pheromones from different chemical classes, such as fatty acids and steroids [41]. Interestingly, ligands binding to allergenic proteins could modify some of the features that contribute to their sensitizing capacity or could have intrinsic immunomodulatory capacities themselves [42], as specifically suggested for Fel d 1 by Herre et al. [43]. Evaluating the contribution of bound ligands to the extent of allergen sensitization and/or allergic response regarding IgE binding is of interest. 

To this end, we aimed to (i) identify the Fel d 1–selected ligand interaction stability using molecular dynamic simulation; (ii) elucidate the refinement of Fel d 1 conformational epitope sites using several computational approaches—we have used various algorithms, and the methods can find the epitope sites in the folding pattern and predict the solvent accessibility score; (iii) discover the impact of the ligand binding interactions on predicted B-cell conformational epitopes of Fel d 1 using computational analysis.

## 2. Results

### 2.1. Molecular Dynamics Simulation (MDS)

The MDS analyses were processed with the Fel d 1 heterodimer and seven selected ligands for 50 ns (nanoseconds), and the docked files were collected from the in silico docking study of Bienboire-Frosini et al. [23]. The conformational stability, folding, and dynamic properties of Fel d 1 were analysed using GROMACS v2019 in the virtual simulation environment.

#### 2.1.1. Backbone Conformation of Fel d 1

**Figure 1:**
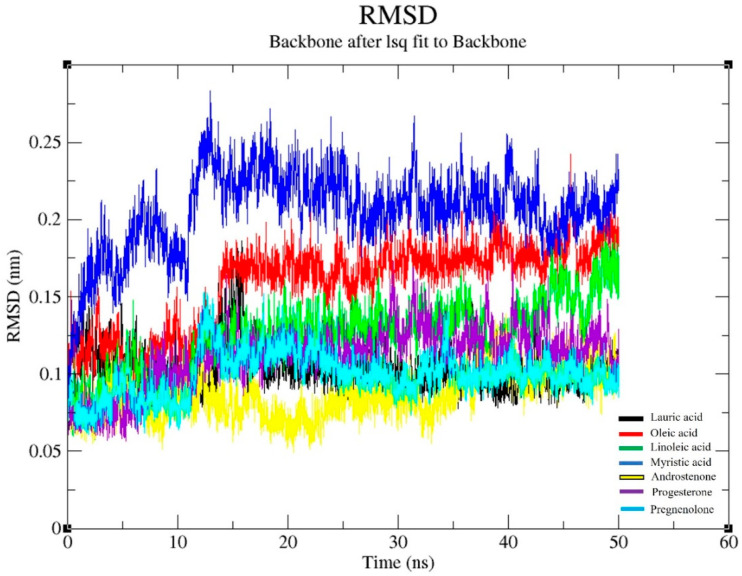
Graphical representation of RMSD for Fel d 1 backbone conformation (Fel d 1 with seven selected ligands). All the Fel d 1 backbone graphs were coloured according to the ligand members in the internal legend.

The RMSD (Root Mean Square Deviation) of the Fel d 1 backbone conformation stability was obtained from the MDS analysis (Figure 1). We observed seven RMSD plots for the conformation of backbones, proteins, and Cα structures. Since the backbone conformations highly corresponded to minimal changes to the structure, we presented only the backbone conformation of Fel d 1 complexed with ligands.

The backbone conformation of Fel d 1–oleic acid, Fel d 1–myristic acid, and Fel d 1–pregnenolone had more structural deviation at the beginning of the simulation for the first 15 ns, but all the structural backbones gained more stability and less deviation during the period between 20–50 ns. Nevertheless, the stable conformations observed for Fel d 1–lauric acid, Fel d 1–androstenone, and Fel d 1–progesterone showed less deviation and were dynamically stable at the position of 0.1 to 0.15 nm (nanometres) of RMSD, except for the first 15 ns. We noticed a sudden surge at 10–15 ns for all the compounds and, subsequently, the peak sliding to the stable RMSD plot, which continued until the end of the simulation. Overall, the RMSD of all protein backbone conformations stayed below 0.25 nm. Interestingly, the Fel d 1 backbone corresponding to the lauric acid complex (black) showed a more dynamically steady RMSD and less deviation because of the compact amino acid contribution in the protein.

#### 2.1.2. Fel d 1–Ligand Complex Dynamics

**Figure 2:**
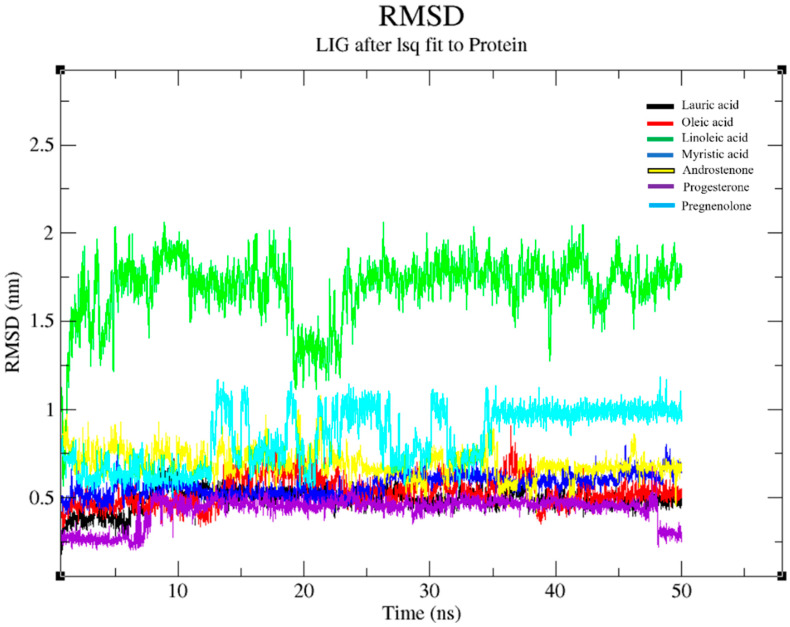
Graphical representation of RMSD for the Fel d 1–ligand complexes. All the Fel d 1–ligand complex graphs are coloured according to the ligand members in the internal legend. The timescale (50 ns) is shown in nanoseconds and the RMSD in nanometres (nm).

The RMSD values of the Fel d 1–ligand complexes were obtained by MDS analysis. We employed the values of the RMSD plots of the seven selected ligand complexes with Fel d 1 using the QtGrace tool (Figure 2). The present results show that the ligand-bound Fel d 1 complex underwent 50 ns of dynamic conformational analysis.

We observed more dynamic stability and less structural deviation for the Fel d 1–lauric acid (black), Fel d 1–myristic acid (blue), and Fel d 1–progesterone (violet) complexes than the Fel d 1–linoleic acid (green) and Fel d 1–pregnenolone (cyan) complexes. Notably, the Fel d 1–lauric acid and Fel d 1–progesterone complexes displayed a very low RMSD plot, below 0.6 nm, compared to other compounds. Fel d 1–lauric acid showed a sudden surge at 10 ns (beginning of the simulation) and remained stable with high compactness throughout the simulation. The ligand binding site of the Fel d 1–lauric complex was not interrupted, and dynamically reliable conformation occurred within the binding pocket. The Fel d 1–oleic acid (red) and Fel d 1–myristic acid (blue) complexes both depicted less deviation, and the RMSD plot adjacent to them was lower than 0.8 nm. Likewise, Fel d 1–linoleic acid displayed more deviation from the plots but showed dynamically stable conformation from 30 ns to 50 ns. Furthermore, we predicted the separate dynamic RMSD plot for each complex with the running average line for a better understanding of the plots. A graphical representation of the unique ligand complexes’ RMSD plots is displayed in the Supplementary File (Figure S1).

For the steroids, Fel d 1–androstenone (yellow) showed better stability and less deviation compared to the pregnenolone complex. Fel d 1–progesterone showed very reliable conformation from 10 ns to 45 ns. The plot showed reduced deviation at 45 to 50 ns, and this may be likely to decrease in RMSD for longer simulations. Regarding the Fel d 1–pregnenolone complex, a more stable conformation was noticed at the end of the simulation, from 35 ns to 50 ns, whereas much deviation and conformational changes were noted from 15 ns to 35 ns. The Fel d 1–lauric acid, –oleic acid, –androstenone, and –progesterone complexes revealed stable conformations of the Fel d 1 backbone and Fel d 1–ligand complexes.

#### 2.1.3. Radius of Gyration (Rg) Analysis

**Figure 3:**
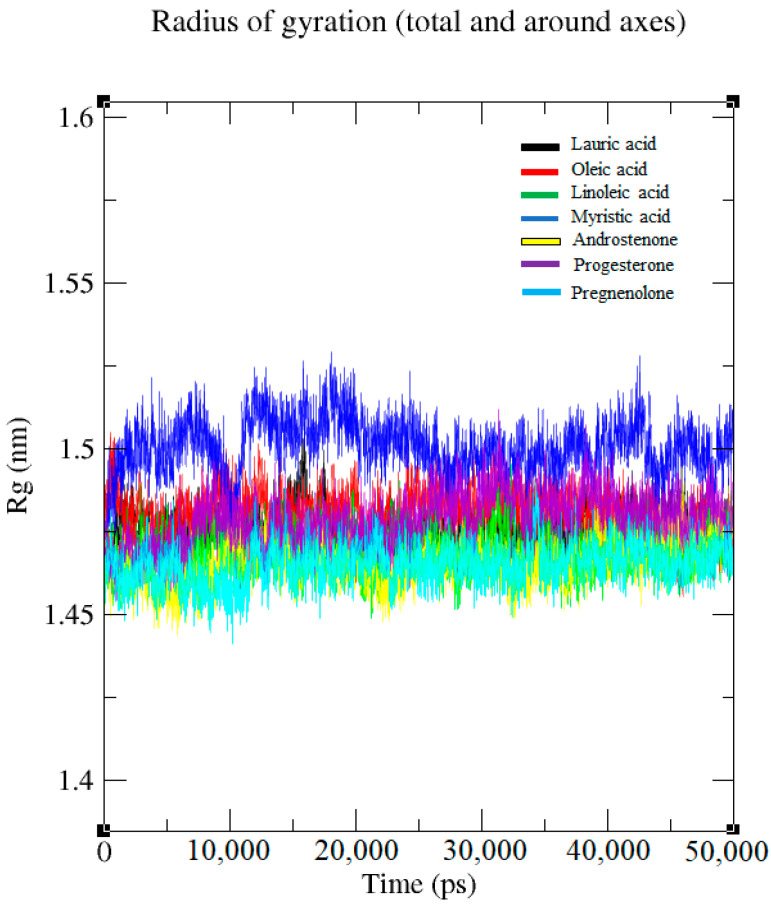
Fel d 1–ligand complexes showing high stability and compactness in radius of gyration (Rg) plots.

The results of the Rg analysis reveal the folding stability and structural compactness of the Fel d 1 corresponding to the ligands (Figure 3). The topology and MDS files were used to predict the gyration plot. A relatively steady value of Rg was maintained in all the structures except Fel d 1–myristic acid, which had partially acquired compactness compared to the other compounds. The Fel d 1–myristic acid complex may have shown protein unfolds in the simulation so that the Rg value and plots were changing over time. Interestingly, we studied the Rg compactness of the Fel d 1–lauric acid, –oleic acid, –androstenone, and –progesterone systems and detected reasonably invariant Rg values. Furthermore, the protein structures remained very stable below 1.5 nm of Rg and displayed reliable compactness until 50 ns at 300 K.

#### 2.1.4. Hydrogen Bond Interaction Analysis

**Figure 4:**
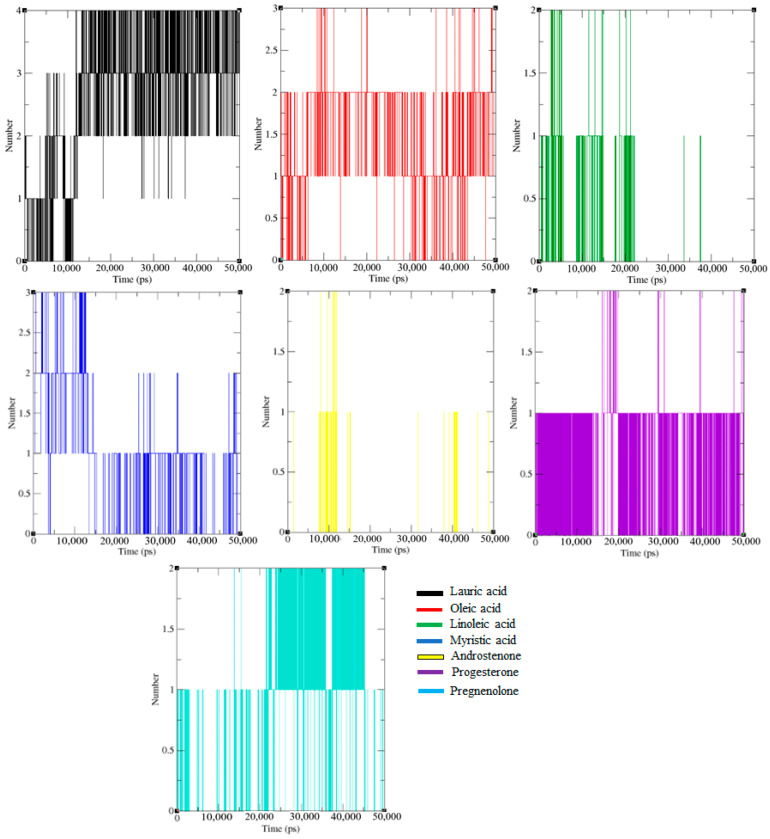
The intermolecular H-bond interaction plot of seven selected ligand-bound complexes with the protein Fel d 1. All the Fel d 1–ligand complex graphs are coloured according to the ligand members in the internal legends.

The insight into the binding phenomenon of Fel d 1–ligand complexes was monitored for 50 ns using hydrogen-bond (H-bond) interaction analysis, and the H-bond interactions are plotted in Figure 4. The MDS analysis revealed that the Fel d 1–ligand complexes showed strong bonding interactions throughout the 50 ns simulation. Furthermore, Fel d 1 had more interactions with the lauric, oleic, and myristic acids.

Interestingly, Fel d 1–lauric acid and Fel d 1–oleic acid displayed six and five H-bond interactions, respectively. These H-bond interactions were formed with critical ligand binding residues and showed an excellent binding affinity and stable interaction towards the active site of the Fel d 1. The androstenone and progesterone complexes had partial H-bond interactions. In addition, a minimum number of H-bond interactions was detected in the Fel d 1–linoleic acid and Fel d 1–pregnenolone complexes. The combined form of the H-bond interaction plot is shown in Figure S2 for the Fel d 1–ligand complexes.

### 2.2. Computational Fel d 1 Epitope Prediction

In this sequence analysis study, the prediction and identification of surface-exposed or -accessible residues are significant for the understanding of functionally exposed residues in Fel d 1 polypeptide chains. Using a ConSurf analysis, the predicted functionally conserved and exposed residues of Fel d 1 chain 1 are C3, Y21, N37, A38, L41, K42, C44, D46, T50, D53, K54, K63, I64, and C70, and the residues of chain 2 are V1, E5, T6, G20, A34, T35, E3, K44, Q46, Y49, E51, G53, R56, D59, S70, and C73. Some other exposed residues are located within the average conservation scale, which was predicted according to the neural network algorithm. The surface-accessible residues predicted on several sites (17–23; 50–55; 103–115; and 137–140) of the Fel d 1 dimer, and similar sites, were predicted for both polypeptide chain 1 and chain 2 (Figure S3A–C). Significantly, the amino acid residues (50–55) had the highest score and showed extremely accessible residue sites in chain 1. Likewise, the glycosylation site N103 and the -104ATEPE108- sites contained highly accessible residues in chain 2.

#### 2.2.1. Antigenic Determinant Site Prediction

The specificity of antigenic peptides was calculated based on the physicochemical properties of experimentally determined epitopes using the Kolaskar and Tongaonkar methods [44]. The antigenic plot was predicted for the Fel d 1 dimer (Figure S4A), polypeptide chain 1 (Figure S4B), and chain 2 (Figure S4C). In addition, all antigenic peptides are shown in Table 1, which were predicted according to the threshold score from various computational servers. Among these results obtained from various approaches, similar antigenic sites were identified for chain 1 of Fel d 1, such as “-21YVEQVAQYKALPVVLEN37-” and “-46DAKMTEEDKE55-”. Additionally, chain 2 of Fel d 1 had putative antigenic sites, which were “-91NELLLDLSLT100-” and “-114KIQDCYVEN122-”. Some other antigenic sites were predicted near the disulfide bridge and N/C-terminal site of the rFel d 1 protein. All the servers provided a score superior to 0.75 except the AlgPred server, which predicted only a single peptide of 36–59 residues.

#### 2.2.2. B-Cell Epitope Prediction Using Webservers

**Figure 5:**
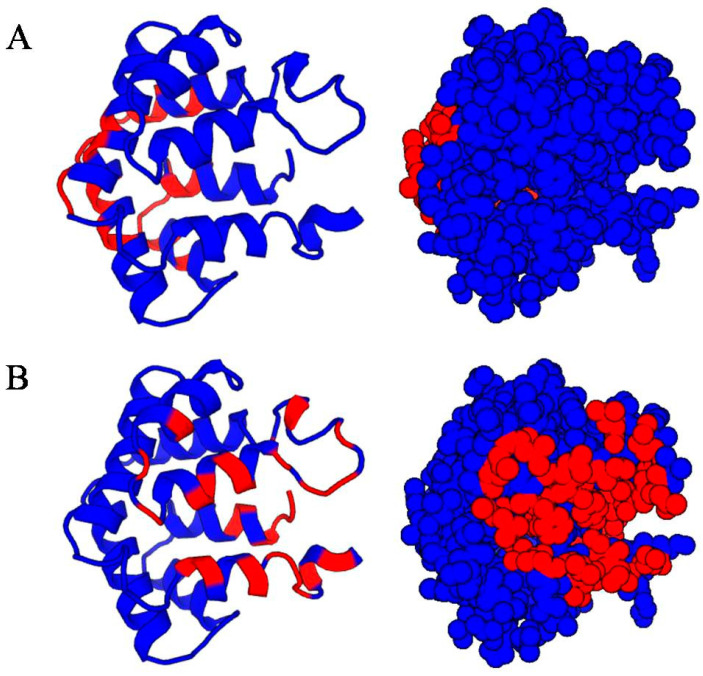
EpiPred analysis of Fel d 1. (**A**) The lateral presence of the epitope sites (between H3–H4) is marked as red coloured with the space fill model. (**B**) The entrance of the epitope sites (H1 and *N*/*C*-terminal) is marked as red coloured with a space fill model.

The sequence and structures of Fel d 1 (dimer and chains 1/2) were employed in several B-cell epitope prediction servers with various algorithms, and the predicted conformational epitope sites were tabulated with distinct threshold values (Table 2 and Table S1). Significantly, (i) DiscoTope and Ellipro were identified as similar conformational epitope sites “-50TEEDxE55-”, where E51 was predicted as an IgE-binding epitope in previous reports [37]. (ii) The CBTOPE algorithm, which calculates antibody-interacting residues with 85% accuracy, predicted that the major conformational epitope sites were localized in the N terminal regions using the SVM method. Particularly, the epitope sites E20, E23, E51, and K54 were obtained with this algorithm. (iii) Some important B-cell epitope sites, L12, T15, T17, P18, E20, R39, N43, D46, E51, K54, D117, Y119, E121, N122, and -138SSSxD142-, were predicted in Fel d 1 using the EpiPred server. The presence of epitope sites in the entrance (helix 1 and N/C terminal) and lateral (helix 3 and 4) was predicted and represented as a space-filled model (Figure 5A,B). The surface accessibility of the protein calculated the sensitivity and specificity score.

(iv) Likewise, the CEP server predicted conformational epitopes from the spatial configuration and structural fitting of the 3D structure. The results show that the similar L12, T17, D46, E51, and K54 were predicted to be highly accessible surface residues in the antigenic site. (v) The exact B-cell epitope residues “T17, P18, E20, E23, R39, N43, D46, E51” were predicted using the epitope prediction method by employing support vector regression (EPSVR). (vi) The BEPro results show that the residues were selected around the 0.85- to above 1.0-threshold level (Figure S5A,B).

The top conformational residues were observed at the 1.0 threshold. The sequence range of 46–56 showed the best conformational site in chain 1. The 35–52 range and N/C-terminal sites displayed higher score limits in chain 2 of Fel d 1. Overall, the conformational epitope residue pattern and scoring plot were predicted depending on the antibody and antigen binding sites in the Fel d 1 dimer and native monomer forms.

#### 2.2.3. Fel d 1-IgE Interactions

Conversely, we identified and refined the conformational epitope sites by assembling the dimers (2ejn_A and 2ejn_B) and polypeptide structures (chain 1 and chain 2) of Fel d 1 with two different experimentally determined IgE antibodies (mAbC48 and nAb) (Table S2). We obtained the structural model for mAbC48 and employed it in the interaction analysis. The structural dimers and monomers of Fel d 1 interacted individually with the modelled mAbC48 and nAb (PDB ID: 5VYF) structures. We observed several H-bonds and hydrophobic interactions between Fel d 1 and IgE heavy/light chain structures. The dimers of Fel d 1_A and B had maximum H-bond interactions in chain 1 (Helix1–Helix4), with both antibodies. More precisely, the residues of Fel d 1 polypeptide chain 1 that were involved in the mAbC48 interactions were R8, D19, E20, E23, E36, and R39. Conversely, chain 2 residues that were involved in the mAbC48 interactions (K114, Y119, and D130) were localized at the highly accessible surface residues. Regarding the nAb interactions, other Fel d 1 conformational residues were involved. Interestingly, we found that dimer B showed H-bond interactions with the antigenic sites of Fel d 1, such as D19, K48, E51, K114, Q116, and D117, compared with the mAbC48 structure. Additionally, chains 1 and 2 showed similar H-bond interactions with the nAb structure. The results were obtained according to the protein–protein docking interactions. On the other hand, we performed a sample test of a 50 ns MDS analysis for the lauric acid (LAU) with the mAb (heavy chain)-bound Fel d 1 subunit A complex. Stable structural conformations were observed for the receptor complex, but when it interacts with ligands, it showed few structural deviations from 10 to 30 ns, followed by consistent stability in the complex from 40–50 ns (Figure S6). However, the MDS should be extended to better assess and study the protein/ligand/mAb complex conformation in depth for future analysis.

### 2.3. Conformational Epitope Interaction on Ligand Binding Site

**Figure 6:**
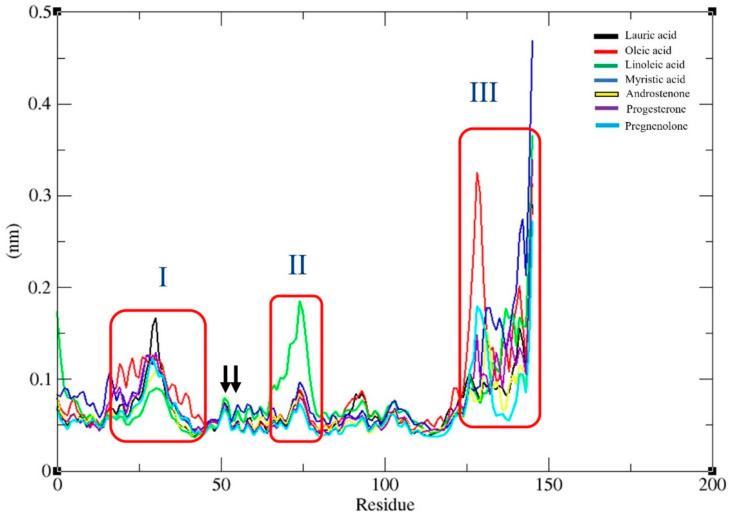
Root mean square fluctuation (RMSF) graph for Fel d 1 and ligand-bound complexes resulting from the 50 ns timescale of MDS analysis. (I) Fel d 1–lauric and Fel d 1–oleic acid complexes; (II) Fel d 1–linoleic acid complex; (III) Fel d 1–oleic acid, –myristic acid, and –pregnenolone complexes. The arrows indicate the E51 and E52 sites in the RMSF plot. All the Fel d 1–ligand complex RMSF graphs are coloured according to the ligand members in the internal legends.

Examining the residue-wise fluctuation through RMSF may suggest the important core residues for the RMSD value fluctuations. Therefore, the RMSF values for the Fel d 1–ligand complexes were plotted and are presented in Figure 6. The highest RMSF peaks represented the ligand-induced structural fluctuations in the computationally predicted epitope sites of Fel d 1. The RMSF plot of the Fel d 1–lauric and Fel d 1–oleic acid complexes (I) showed notable fluctuations on the epitope site of “-27QYKALPV33-” in the Fel d 1 structure. Furthermore, Fel d 1–linoleic acid (II) showed more fluctuations on the epitope site of “-66TSPLCVKMAETC77-” (*C*-terminal of chain 1 and *N*-terminal of chain 2 connecting the site). The Fel d 1–oleic acid, –myristic acid, and –pregnenolone complexes (III) showed more fluctuations on the epitope site of “-126SRVLDGLVM134-”, and all the other ligands showed residual fluctuations at the C-terminal epitope site of “-138SSSKSCMG145-”.

In addition, we predicted the antigenic determinant site and B-cell epitope sites of “-46DAKMTEEDKE55-” using various servers. Specifically, at the E51 and E52 sites (see arrows in Figure 6), we noticed a very slight change in the structural conformation due to the interactions with lauric and linoleic acids, as well as progesterone. Additionally, there were no residual fluctuations observed on the “Y21” conserved sites of Fel d 1 except for the lauric acid interaction. Fel d 1–oleic acid showed ligand-induced residual fluctuations on the sites of P18, D19, E20, Y21, V22, and E23, and Fel d 1–myristic acid had fluctuations on the corresponding residues of T15 and T17, which correspond also to the IgE binding conformation epitopes reported by Tasaniyananda et al. [37]. Fel d 1–oleic acid and Fel d 1–myristic acid showed fluctuations on the “-16GTPDE20-” site. All the ligand complex interactions showed more fluctuations on the epitope site of “-138SSK140-” except the Fel d 1–pregnenolone complex. However, overall, most of the ligand binding residues showed very low residual fluctuations (<0.1 nm) during the interaction with the seven selected ligands, which means very stable interactions were observed. The ligand binding residues were validated from the previous docking analysis [23].

### 2.4. Binding Free Energy Validation

The Fel d 1–ligand complexes showed potential binding over the 50 ns of MDS, which was perceived by the binding-free energy and stable conformations. Analysing the molecular interactions between Fel d 1 and the ligand complexes at a time scale of 50 ns using the g_mmpbsa program (Table S3) was validated. The best-fit binding free energy was computed for Fel d 1–lauric acid (−177.327 ± 19.989 kJ/mol), Fel d 1–oleic acid (−121.717 ± 19.408), Fel d 1–myristic acid (−248.775 ± 23.314), and Fel d 1–progesterone (−124.483 ± 10.464). Likewise, considerable values of non-polar attractions (van der Waals) were observed for the lauric, oleic, and myristic acids and the progesterone complex. The steroid molecules showed very low energy during electrostatic interactions. Furthermore, Fel d 1–linoleic acid, Fel d 1–androstenone, and Fel d 1–pregnenolone displayed the minimum binding-free energy of −104.941 ± 36.888, −69.937 ± 16.538, and −61.160 ± 9.291, respectively, towards the active site residues throughout the simulation time, which suggests they exhibit the least amount of binding compared to the other compounds.

## 3. Discussion

An allergen is a foreign substance that can cause an allergic reaction, classified as type 1 hypersensitivity, notably via the triggering of an unwanted Th2-biased immune response and the production of IgE antibodies in sensitized individuals [45]. The abundance and stability of immunogenicity are important characteristics of allergens [46]. Notably, environmental allergens play a significant role in asthma and allergy development. Healthy, sensitized, or allergic individuals certainly have different threshold levels of sensitization associated with an increased risk of disease and/or sensitization [47]. It is believed that IgE evolved in mammals as a first line of defence against pathogens [48]. To understand immune responses to allergens, it is necessary to know the features of the IgE-binding epitopes of a particular allergen [49]. Additionally, immunoinformatics converts large-scale immunological data using computational approaches to attain meaningful interpretations towards the development of epitope-based vaccines [50]. The identification of epitopes has been performed and reported using several approaches such as (i) epitope mapping, studied by monoclonal antibodies against the native and reduced/alkylated major cat allergen Fel d 1 [35]; (ii) the overlapping of synthetic peptides [51]; (iii) predicting IgE-binding epitopes through an allergen surface comparison using SPADE and structure-based epitope prediction methods [28,52]; (iv) measuring the binding affinity of epitopes with HLA-DRB1 alleles using artificial neural network methods [53]; and (v) identifying solvent-exposed IgE epitopes from the X-ray crystallographic structure and the IgE-binding epitopes of the major cat allergen Fel d 1 [8,37].

In the present study, we evaluated the binding stability of Fel d 1 complexed with seven selected ligands using the molecular dynamics simulation (MDS) analysis. The backbone conformation of Fel d 1 showed a more stable RMSD plot, corresponding to lauric acid, androstenone, and progesterone. Interestingly, the Fel d 1 backbone associated with lauric acid showed less deviation in the backbone structure, which revealed the compactness of amino acid contributions in Fel d 1 (Figure 1 and Figure 3). Similarly, the RMSD of the Fel d 1–ligand complexes displayed stable conformations except for the linoleic acid and pregnenolone plots, which showed more deviation than the other complexes (Figure 2). The Fel d 1–lauric acid, Fel d 1–oleic acid, Fel d 1–myristic acid, and Fel d 1–progesterone complexes had very reliable conformations, but Fel d 1–lauric acid and Fel d 1–oleic acid had more hydrogen bond interactions than the other ligands (Figure 4). The RMSD graph of Fel d 1–lauric acid maintained less deviation and remained stable throughout the simulation. Therefore, our previous findings based on an in-vitro spectrofluorimetric ligand-binding assay and classical docking of Fel d 1–lauric acid are validated by the present dynamic simulation study [23]. Overall, the stability of the Fel d 1–ligand (lauric acid, linoleic acid, and progesterone) complexes is outstanding. Of note, other proteins involved in chemical communications, such as OBPs from the lipocalin family, also displayed highly stable structural conformations when complexed with their allergenic ligands [54,55,56].

On the other hand, a previous report suggested the reduced and alkylated Fel d 1 displayed a selective effect on monoclonal antibody binding [35]. As a result of the reduction and alkylation experiment, these authors suggested that the conformational epitope sites are formed by four significant Fel d 1 peptide regions. Additionally, we elucidated the surface-accessible/exposed residues and conserved residues in our previous study [22]. It is important to predict the B-cell conformational epitope sites of Fel d 1 to analyse the computational modification/inhibition of the molecular interactions and to implement advancements in immunotherapy. Therefore, the antigenic determinant sites were analysed by several servers using the Fel d 1 monomers and dimers (Table 1). The results show that Fel d 1 had several accessible/exposed residues and antigenic sites in the *N*/*C*-terminal regions. The native Fel d 1 peptides, such as 1–16 and 60–70 from chain 1 and 1–14 and 43–56 from chain 2, showed inhibitions on antibodies directed against a conformational epitope formed by these four regions [35]. Similar results were shown for the birch pollen profilin allergen [57], and the *N*- and *C*-terminals of the Can f 1 protein form were also conformational epitope sites [58]. A higher residual conservation of Fel d 1 was observed for the conformational epitope sites Y21, N37, D46, T50, D53, K54, K63, and C70 of chain 1 and E32, K38, Q40, C42, and E45 of chain 2 with the mouse ABP homologue. The conformational epitopes of a protein are predicted by determining surface accessibility, residual propensity, spatial proximity, and contact with the Ab. We predicted the B-cell conformational epitope sites of Fel d 1 and presented the cumulative sites by indicating the ligand binding sites and IgE-binding epitope sites, as seen in Table 2. Furthermore, the predicted conformational sites were confirmed by the Fel d 1–IgE complex interactions (Table S2). Additionally, we compared the conformational epitope sites with literature references. We observed several conformational sites on the Fel d 1 monomers and dimers, which corroborated a previous report on Fel d 1–IgE binding [37].

We detected some conformational changes in the residues due to ligand binding on the epitope sites, which was confirmed by the RMSF plot. The lauric and oleic acid exhibited notable fluctuations on the predicted epitope sites in the *N*- and *C*-terminals, but the oleic acid alone triggered more fluctuations from S126 to M134. Hence, the Fel d 1–ligand interactions may affect the conformational epitope sites and lead to significant structural changes in the epitope sites. The MDS results reveal that putative ligand interactions with the predicted B-cell epitope sites influenced the structural conformation of the protein. According to a previous study, non-specific lipid transfer proteins also had different IgE-binding properties resulting from fatty acid-binding alterations and residual conformation variations [59]. Consequently, the Fel d 1’s specific epitope sites might show different residual conformations while binding antibodies and ligands. As previously reported, chain 1 reflected the established evolution of the family Felidae, whereas chain 2 orthologs varied between species due to the variability of T82-D89 residues [60]. Furthermore, the folding pattern of the polypeptide chains results in an antiparallel orientation. This asymmetry plays an important role in the recognition of each monomer (chains 1 and 2) in terms of immunological response [61]. In addition, the correct orientation of the chains is important for determining the antigenic site of IgE [62]. Moreover, the cavity volume and the selective ligand binding response of subunits A and B also exhibited few variations in residues. The current results confirm our previous study [23], hence strongly suggesting that Fel d 1 can serve as a shuttle by transporting fatty acids and steroids and plays a crucial role in the cat’s chemical communications. The slight effect of ligand binding on conformational epitopes and IgE binding was predicted by the present in silico study.

## 4. Materials and Methods

### 4.1. System Configuration

All the computational analyses were performed on high-performance GPU workstations running CentOS V.7.6 Linux and the Windows operating system. The hardware specifications of the workstation (Model: LVX-1 × RTX-2080Ti-i9) included a powerful Intel Core i9-9920X processor with 1 GPU RTX-2080Ti (11 GB-RAM-DDR6) and 32 GB RAM, running with super-fast, boot-home 1 × M2-1 TB and 2 × 8 TB independent hard drives. The workstation passed all the validation tests to comply with the GPU-certified *Linuxvixion* system (Madrid, Spain).

### 4.2. Dataset Collection

The X-ray crystallographic structure of recombinant Fel d 1 (rFel d 1) was obtained from the Protein Data Bank (PDB) (https://www.rcsb.org/ (accessed on 6 July 2022)) and the polypeptide sequences of chain 1 (P30438.2) and chain 2 (P30440.1) were retrieved from the UniProt database (https://www.uniprot.org (accessed on 6 July 2022)). Furthermore, the 7 selected ligands (lauric acid: CID_3893; oleic acid: CID_445639; linoleic acid: CID_5280450; myristic acid: CID_11005; androstenone: CID_6852393; progesterone: CID_5994; pregnenolone: CID_8955) were retrieved from the previous classical docking files of Fel d 1 [23], and the files were separated as protein and ligand alone using the Discovery studio visualizer (v.21.1.0.20298) for the simulation process. All the ligands were registered in Sybyl MOL2 format with the addition of polar charges to the structures, and the protein subunit was registered in PDB (.pdb) format. In the present study, we used this sequence and structural information of Fel d 1 in all the computational methods.

### 4.3. Molecular Dynamics Simulation (MDS)

Molecular dynamics simulation is a very powerful toolbox in the modern generation of molecular modelling. It enables us to understand the precise details of the structure and dynamics of the motion of individual atoms [63]. In the present study, the Fel d 1–ligand complexes were simulated for 50 nanoseconds (ns), equal to 50,000 ps. The scope of MDS has greatly expanded in several fields, and it has become more accurate with better force fields [64]. The molecular toolkit GROMACS was updated with high performance on algorithm optimization and enables long simulations of large systems [65].

#### 4.3.1. Protein Preparation

The MDS was carried out for the X-ray crystallographic structure of Fel d 1 with seven selected ligands using the GROMACS (Groningen Machine for Chemical Simulations) v.2019.3 (https://www.gromacs.org/ (accessed on 6 July 2022)) simulation [64,66]. The subunit A of the rFel d 1 structure was obtained from a previous classical docking analysis [23], and the calcium ions with HETATM were deleted for the MDS analysis. Refinement was performed by processes such as the verification of bond orders, the removal of water molecules, adding missing residues, and side chain refinement.

#### 4.3.2. Generation of Topology and Solvation

The protein topology files were generated using the CHARMM36 all-atom force field (2019), which was obtained with the “cgenff_charmm2gmx.py” conversion script from the *MacKerell* lab site (http://mackerell.umaryland.edu/charmm_ff.shtml#gromacs (accessed on 6 July 2022)). The selected ligands were used to generate the topology using the official CHARMM General Force Field server CGenFF (https://cgenff.umaryland.edu/ (accessed on 6 July 2022)). The ligand topology files were combined with the protein and were created through a complex.gro file for the solvation process. The Fel d 1–ligand complexes were immersed in a dodecahedral unit cell shape box with a 1.0 nm distance allowed between the edge of the box and the Fel d 1 surface. The box contained simple point charge (spc216) water molecules and appropriate counter ions, Na^+^ and Cl^−^, to neutralize the net charge on the system. The system was neutralized by replacing Na^+^ counter ions instead of Cl^−^ ions in the topology file for rebalancing the charges.

#### 4.3.3. Energy Minimization and Equilibration

The initial energy minimization process was started with the complex system for 1000 steps of the steepest descent algorithm. The potential energy was obtained by the minimization of the complex with ions, and this ensured the system had no steric clashes and inappropriate geometry. The molecular bond lengths and geometry were constrained by the holonomic constraints (LINCS) [67] algorithms. After that, the equilibration was conducted in two phases, NVT (temperature) and NPT (pressure); both equilibrations were run for 100 ps with the position of the protein–ligand complex restrained, and the temperature and pressure of the whole system stabilized [68]. The thermostat coupling was set for the reference temperature of 300 K by the Berendsen thermostat, with the incorporation of a coupling time of 0.1 ps. The long-range electrostatic interactions were calculated using the coulomb type of Particle-Mesh Ewald (PME) method for biomolecular systems [69,70].

#### 4.3.4. Production of MDS

After the completion of the two equilibration phases, the simulation system was well equilibrated with the desired NVT and NPT parameters. In continuation, the pre-equilibrated system of Fel d 1–ligand complexes was subjected to the 50 ns (i.e., 50,000 ps) production of the MD simulation at intervals of 2 femtoseconds, and the trajectories were saved for further analyses. The pressure coupling of the 1.0 bar reference pressure was applied using Parrinello–Rahman along with the periodic boundary conditions. We then performed a recentre and rewrap of the molecules within the unit cell to recover the desired rhombic dodecahedral shape. For the MDS analysis, the complexes were ensured to analyse the RMSD, RMSF, H-bond, and interaction energy (ie) between Fel d 1–ligands using VMD and QtGrace v.026.

#### 4.3.5. g_mmpbsa Analysis

In the present study, the high-throughput binding energy calculation method (ΔG*_residue_* = ΔG*_ele_* + ΔG*_nonpol.sol_* + ΔG*_vdW_* + ΔG*_polar_*) of g_mmpbsa was applied to predict the binding-free energy between the Fel d 1–ligand complexes [71,72,73]. The MMPBSA (molecular mechanic’s Poisson–Boltzmann surface area) method was used to evaluate the relative stabilities of different biomolecular structures [74,75]. The g_mmpbsa was developed using GROMACS and APBS (Adaptive Poisson–Boltzmann Solver). The trajectory, topology, and index files were provided as inputs to the g_mmpbsa-free energy decomposition scheme. The results were analysed by the python script (MmPbSaStat.py) file for obtaining whole summary interactions such as binding-free energy, van der Waals, SASA (solvent-accessible surface area), electrostatics, and polar solvation energy.

### 4.4. B-Cell Conformational Epitope Prediction

#### 4.4.1. Literature Mining

The B-cell epitopes and the allergenic determinant peptides were collected from earlier reports. The collected linear and discontinuous epitopes were classified, and we removed redundancies using the CD-Hit server (https://github.com/weizhongli/cdhit-web-server (accessed on 30 November 2018)). The reported epitope sites, features, and residual positions are presented in Table 3.

#### 4.4.2. Physio-Chemical Property Analysis

The dimeric and monomeric sequences of Fel d 1 were applied to predict physico-chemical properties, such as accessible surface residues, using the Emini surface accessibility scale [76] and protscale (https://web.expasy.org/protscale/ (accessed on 28 November 2018)) methods. In addition, the sequence dataset was validated through the IEDB webserver (http://tools.iedb.org/bcell/ (accessed on 28 November 2018)) for hydrophilicity (Parker) and protein flexibility (Karplus and Schulz). Furthermore, the ConSurf analysis was performed for the prediction of functionally surface-exposed residues (http://consurf.tau.ac.il/2016/ (accessed on 28 November 2018)) using the Fel d 1 sequence with ABP homologues (Supplementary Figure S7A–D).

#### 4.4.3. Antigenic Determinant Peptide Prediction

The Fel d 1 sequence and structures were used to predict the antigenic peptide and antigenicity score of proteins using several methods, such as (i) the Kolaskar and Tongaonkar antigenicity method from IEDB (accessed on 11 October 2018); (ii) SVMTriP (accessed on 18 October 2018); (iii) the prediction of allergenic sites in proteins (AlgPred) (accessed on 6 November 2018); (iv) protein antigenicity prediction based on the five machine-learning algorithm Antigenpro (accessed on 6 November 2018); and (v) EMBOSS (accessed on 15 October 2018).

#### 4.4.4. Computational Prediction of B-Cell Epitopes

Additionally, a list of the computational servers is provided for the prediction of conformational epitope sites in proteins (Table 4).

These computational programs were used to refine and revalidate the B-cell conformational epitopes in Fel d 1. Notably, the CEP algorithm provides a web interface to compute the percentage of accessibility of residues and the spatial distance cut-off to predict antigenic determinants [27]. The CBTOPE server can predict epitope sites from the primary sequence of antigens using a support vector machine (SVM) with an accuracy of more than 85% and an area under the curve of 0.9. Similarly, EPSVR predicts the antigenic epitope sites using the support vector regression method [77]. The EpiPred algorithm calculates the epitope sites by knowledge-based asymmetric Ag–Ab scoring and a combination of geometric fitting of the protein [78]. Interestingly, the ElliPro method predicts epitope sites associated with a score called a protrusion index (PI) value [79] using protein 3D structures, which predicts linear and conformational epitopes.

#### 4.4.5. Molecular Interactions between Fel d 1-IgE Antibody

The heavy (H) and light (L) chain sequences of mAbC48 were collected [37] and submitted to the PS_2_V2 (http://ps2v2.life.nctu.edu.tw/ (accessed on 10 October 2018)), RaptorX (http://raptorx6.uchicago.edu/ (accessed on 15 October 2018)), and PIGsPro (accessed on 5 November 2018) servers for the modelling of the antibody domain using the canonical structure model. Furthermore, we selected a good model through the structural validation analysis, which was carried out using the ProFunc server. Similarly, the structure of nAb was collected from PDB (ID: 5VYF), and the 1PUO structure was removed from each chain. The dimer and monomer chains of Fel d 1 were docked to the mAb and nAb structures, respectively, using the ClusPro server (https://cluspro.bu.edu/login.php (accessed on 5 November 2018)). This server provides the best-fit ranking of docked poses, and it is allowed to perform the antibody mode for the Ag–Ab complexes.

## 5. Conclusions

This study provides new insights into the respective epitope sites on B-cells and their conformational changes during ligand interactions, which show that ligand interactions synergistically inhibit distinct epitopes of Fel d 1. Therefore, it is interesting to evaluate the interaction between the IgE of cat-allergic patients and Fel d 1-bound ligands using in vitro analyses. Due to the prevalence and frequency of the major cat allergen Fel d 1 in a cat owner’s house, as well as in public environments, it is necessary to develop strategies for “neutralizing” this allergenic risk. These would need to control the binding of specific ligands to Fel d 1. In this context, a noteworthy publication reported techniques for the removal of endogenously bound ligands and, if necessary, their replacement with lipids of known composition of several allergens [80]. Future studies should also investigate the impact of specific ligand binding on the Fel d 1 sensitization processes, as previously hypothesized [42].

## Figures and Tables

**Table 1:** The antigenic determinant peptides were predicted using the Fel d 1 sequence. Similar antigenic peptides are coloured the same way and indicated in chain 1 (bold) and chain 2 (italics).

Tools and Webserver Links	Score	Antigenic Peptide_2EJN
Predicted Antigenic peptides (http://imed.med.ucm.es/Tools/antigenic.pl (accessed on 11 October 2018))	1.0475	CPAVKRDVDLFL PDE**YVEQVAQYKALP**VVLEN ILKNCVD **KMTEEDKE** NALSLLDKIYTSPLCVKMAETCPIFYDVFFAV *NELLLDLSLTK* *KIQDCYVEN* LISRVLDGLVMT
SVMTrip (http://sysbio.unl.edu/SVMTriP/ (accessed on 18 October 2018))	1	NCEICPAVKRDVDLFLTGTP CV**DAKMTEEDKE**NALSVLDK *NELLLDLSLT* *DCYVEN*GLISRVLDGL
AlgPred (https://webs.iiitd.edu.in/raghava/algpred2/ (accessed on 6 November 2018))	0.29578	ENARILKNCV**DAKMTEEDKE**NALS
SCRATCH (http://scratch.proteomics.ics.uci.edu/index.html (accessed on 6 November 2018))	0.792789	**QYKALP** CV**DAKMTEEDKE**N SLTKVNATEP TISSSKDCMGEHHHHHH
EMBOSS (http://imed.med.ucm.es/cgi-bin/emboss.pl?_action=input&_app=antigenic (accessed on 15 October 2018))	1.11–1.89	PAVKRDVDLFLT DE**YVEQVAQYKALP**VVLENA LKNCVDA *ELLLDLSLT*KV *IQDCYVEN*G ISRVLDGLVMTT

**Table 2:** B-cell conformational epitope sites of Fel d 1. The sites were predicted in the Fel d 1 dimer and polypeptide chains based on several algorithms. The putative ligand-binding residues and reported IgE-binding epitopes are highlighted in bold and italics, respectively.

Servers and Links	Threshold	Predicted Conformational Epitope Sites
		PDB ID: 2EJN
DiscoTope (http://tools.iedb.org/discotope/ (accessed on 5 October 2018))	−7.7	*T17*, D19, A47, T50, *E51*, E52, D53, E55, A74, T105, E106, P107, G123, K141, M144
ElliPro (http://tools.iedb.org/ellipro/ (accessed on 28 November 2018))	0.5	K29, A30, L31, P32, V33, T50, *E51*, E52, D53, E55, V71, K72, M73, A74, E75, N103, A104, T105, E106, P107, T110, S139, S140, K141, D142, C143, M144, G145
CBTOPE (https://webs.iiitd.edu.in/raghava/cbtope/submit.php (accessed on 30 November 2018))	−0.3	E1, I3, D19, *E20*, **Y21**, V22, *E23*, Q24, V25, A26, Q27, Y28, A30, T50, *E51*, E52, D53, *K54*, E55, N56, S59, **L61**, D63, L69, C70, V71, K72, M73, A74, **F80**, **Y81**, N91, A111, **M112**, K113, K114, **I115**, Q116, D117, C118, **Y119**, E121, **S138**
EpiPred (http://opig.stats.ox.ac.uk/webapps/newsabdab/sabpred/epipred/ (accessed on 16 October 2018))	−3.7	D11, *L12*, **F13**, *T15*, G16, *T17, P18*, D19, *E20*, *R39*, I40, *N43*, C44, *D46*, A47, K48, T50, *E51*, E52, D53, *K54*, E55, N56, L58, S59, K114, D117, **Y119**, E121, N122, **D130**, **G131**, **M134**, **S138**, S139, S140, D142
CEP (http://196.1.114.49/cgi-bin/cep.pl (accessed on 10 October 2018))	≥72%	I2, C3, P4, R8, *L12*, G16, *T17*, *P18*, *D46*, A47, K48, T50, *E51*, E52, *K54*, S67, P68, V71, K72, **F80**, T105, K114, **I115**, Q116, **D130**, **S138**, S139
EPSVR (http://sysbio.unl.edu/EPSVR/ (accessed on 26 November 2018))	0.638	G16, *T17*, *P18*, D19, *E20*, *E23*, A26, A30, L31, L35, *R39*, I40, *N43*, *D46*, *E51*, E75, **D82**, **F85**, **N89**, G90, N91, **L94**, L97, V120, I125, **G131**, **S138**
BEPro (http://pepito.proteomics.ics.uci.edu/index.html (accessed on 30 November 2018))	0.85–1.0	A30, P32, V33, A47, K48, T50, *E51*, E52, D53, E55, N66, P68, L69, V71, K72, A74, V92, A94, T95, E96, P97, L99, E121, **S138**, S139, S140, D142, G145
BepiPred (https://services.healthtech.dtu.dk/service.php?BepiPred-2.0 (accessed on 26 November 2018))	0.55	P4, K7, R8, D19, *E20*, *E23*, Q24, A26, Q27, K29, A30, L31, P32, V33, K48, T50, *E51*, E52, D53, *K54*, E55, P68, C70, K72, M73, E75, E92, L97, S98, T100, K101, N103, T105, E106, P107, E108, R109, T110, K113, K114, D117, V120, E121, N122, G123, I125, S126, R127, **L129**, **D130**, K141, D142, M144, G145, E146, H148

**Table 3:** The reported peptides and B-cell epitope sites in Fel d 1. The surface-exposed residues are highlighted in bold, which was confirmed by Kaiser et al. [8].

S. No	Article References	Epitope Feature	Chain 1 with Sequence Number	Chain 2 with Sequence Number
1	Kaiser et al., 2003 [8]	Solvent-exposed IgE epitopes	Q27, L31, P32, E36, A47, E51, E52, E55	F15, N19, E22, L23, L27
2	van Milligen et al., 1994 [14]	Linear peptides	VA**Q**YKA**LP**VVL**E**NA-K (25–38) D**A**KMTE**E**DK**E**NALS-K (46–59)	**F**AVA**N**GN**EL**LLDCS-K (15–28)
3	van’t Hof et al., 1993 [35]	Conformational sites in peptides	EICPAVKRDVDLFLT (1–15) LLDKIYTSPLC (60–70)	VKMAETCPIFYDVF (1–14) KKIQDCYVENGLIS (43–56)
4	Tasaniyananda et al., 2016 [37]	IgE-binding conformational epitopes	L12, T15, T17, P18, E20, E23, R39, K42, N43, D46, E51, K54	

**Table 4:** List of computational servers for the prediction of conformational epitope sites in the 3D protein structure of proteins. The relevant methods are provided.

Servers	Methods
DiscoTope	Structure-based antibody prediction.
ElliPro	Ag-3D structure linear and conformational epitope prediction protrusion index (PI).
CBTOPE	This algorithm discriminates the antibody epitope residues and non-epitope residues for a given primary sequence.
EPiPred	Predicting the structural epitopes by knowledge-based asymmetric Ag–Ab scoring.
CEP	Conformational epitope prediction based on antigens and antibodies available in PDB.
EPSVR	Antigenic epitopes prediction with support vector regression.
BEPro	Ag-3D structure based discontinuous B-cell epitope prediction.
BepiPred 2.0	Prediction of potential linear B-cell epitopes. It is based on a random forest algorithm trained on epitopes annotated from antibody–antigen protein structures.

## Data Availability

The findings of this study are embedded within the article as a Supplementary File, and the dataset is available on reasonable request from the corresponding author.

## Supplementary Materials

The following supporting information can be downloaded at: https://www.mdpi.com/article/10.3390/ijms241411685/s1.
