# Supplementary material for: Impact of Semiochemicals Binding to Fel d 1 on Its 3D Conformation and Predicted B-Cell Epitopes Using Computational Approaches
Source: Int J Mol Sci. 2023 Jul 20;24(14):11685. doi: 10.3390/ijms241411685 (PMC10380945; doi:10.3390/ijms241411685)
Supplement: Supplementary file 1 [file ijms-24-11685-s001.zip › ijms-2478157-supplementary.pdf]

Supplementary Files

Table S1. B-cell conformational epitope sites were predicted in Fel d 1 polypeptide chains (Chain 1 and 2) based on several algorithms.

Servers	Threshold	Predicted conformational epitope sites	
		Chain1	Chain2
DiscoTope	-7.7	D19, A47, T50, E51, E52	A4, T35, E36, P37, G53, K71, M74
ElliPro	0.5	K29, 30, L31, P32, V33, T50, E51, E52, D53, E55	V1, K2, M3, E5, N33, A34, T35, E36, P37, T40, S68, S69, S70, K71, D72, C73, M74, G75
CBTOPE	-0.3	E1, I3, D19, E20, Y21, V22, E23, Q24, V25, A26, Q27, Y28, T50, E51, E52, D53, K54, E55, N56, S59, L62, D63, P68, L69, C70	V1, K2, M3, A4, F10, Y11, N21, A41, M42, K43, K44, I45, Q46, D47, C48, Y49, E51, S68
EpiPred	-3.7	D11, L12, F13, T15, G16, T17, P1, D19, E20, R39, I40, N43, C44, D46, A47, K48, T50, E51, E52, D53, K54, E55, N56, L58, S59	K44, D47, Y49, E51, N52, D60, G61, M64, S68, S69, S70, D72
CEP	≥72%	I2, C3, P4, R8, L12, G16, T17, P18, D46, A47, K48, T50, E51, E52, K54, S67, P68	V1, K2, F10, T35, K44, I45, Q46, D60, S68, S69
EPSVR	0.638	G16, T17, P18, D19, E20, E23, A26, A30, L31, L35, R39, I40, N43, D46, D51	E5, D12, F15, N19, G20, N21, L24, L27, I55, G61, T65, S68
BEPro	0.85->1.0	A30, P32, V33, A47, K48, T50, E51, E52, D53, E55, N66, P68, L69	V1, K2, A4, V32, A34, T35, E36, P37, R39, E51, S68, S69, S70, D72, G75
BepiPred	0.55	T71, E72, E73, D74, K75, E76	I83, S84, S85, S86, K87, D88, C89, M90, G91, E92, A93, V94, Q95, N96, T97, V98, E99, D100, L101, K102, L103

Table S2. The molecular interactions between Fel d 1 with antibodies. The dimers (2ejn_A and 2ejn_B) and polypeptide structures (chain 1 and chain 2) of Fel d 1 were docked with experimentally proved two different antibodies (mAbC48 and nAb). The highlighted Fel d 1 residues (red) are involved in the putative ligand-binding function and the predicted epitope sites are in blue colour.

Protein/Polypeptide Chain	Antibody	Members	H-bond (Feld1-Ab heavy chain)	H-bond (Feld1-Ab light chain)	Hydrophobic residues (Ag)
2ejn_A	mAbC48	129	**R8**-Y109<br>**R8**-Y113<br>**D19**-R107<br>**E20**-Y109<br>**E23**-R107<br>**R39**-Y55<br>**R39**-Y62<br>**R39**-D66	**E20**-Y36	**T15**, **G116**, **T17**, **A30**, **L31**, **P32**, **L35**
2ejn_B	mAbC48	133	**R8**-D66<br>**D11**-T60<br>**T15**-N57<br>**D19**-R107<br>**E20**-R107<br>**E23**-R107<br>**Q24**-Y62<br>**K141**-Q69<br>**D142**-K72	**D19**-Y36<br>**E23**-T116<br>**R39**-Y36	**L12**, **G16**, **T17**, **P18**, **V22, Q27**, **E51**
Feld1_Chain1	mAbC48	222	**R8**-Y113<br>**D19**-R107<br>**E20**-Y109<br>**E23**-R107<br>E36-T60<br>**R39**-Y55<br>**R39**-Y62	**T15**-S32<br>**T15**-R108<br>**T17**-R108<br>**E20**-R108	**L12**, **G16**, **Q24**, **Q27**, **P32**
Feld1_Chain2	mAbC48	142	V102-Y113<br>E108-R107<br>**K114**-T60<br>**K114**-D66<br>**Y119-S59**<br>**D130-T31**<br>**D130-K82**<br>**M134-T29**	**N103**-Y36<br>**E106**-S32	**Phe80, Phe84**, **L99**, **N103**, **A104**, **A111**, **M112**, **I115**, **C118**, **I137**,
2ejn_A	nAb	177	K7-Y101<br>**E23**-R54<br>**Q27**-T28<br>**Q27**-S31<br>**Y28**-Y32	**I2**-Y49<br>**E55**-S30<br>**L58**-K50<br>D62-S31	P4, A5, **R8**, **Q24**, **K141**, **D142**
2ejn_B	nAb	107	**D19**-R54<br>**K48**-Y101<br>**E51**-Y32<br>**E51**-T28	**K114**-E55<br>**Q116**-K50<br>**D117**-K50	**D46**, **A47**, M49, **T50**, **K114**
Feld1_Chain1	nAb	104	**E23**-R54<br>**L35**-R54<br>**N43**-S31<br>N37-N57	**K48**-Y91	**V22**, E36, **R39**, **I40**, **A47**, **K48**
Feld1_Chain2	nAb	147	**K114**-E100<br>**C118**-Y101<br>**N122**-Y104	**Y119-K50**<br>**N122**-E55<br>**D130-S53**	**I115**, **D117**, **E121**

Table S3. Validation of Fel d 1-ligand complex interaction using *g_mmpbsa* analysis.

S. No	Protein complex	Binding energy (kJ/mol)	van der waals energy (kJ/mol)	SASA energy (kJ/mol)	Electrostatic energy (kJ/mol)	Polar solvation energy (kJ/mol)
1	Fel d 1-LAU	-177.327 +/- 19.989	-127.787 +/- 11.065	-16.700 +/- 0.719	-163.290 +/- 29.181	158.525 +/- 20.335
2	Fel d 1-OLE	-121.717 +/- 19.408	-171.706 +/- 15.125	-22.701 +/- 0.798	-97.868 +/- 52.809	218.257 +/- 47.640
3	Fel d 1-LIN	-104.941 +/- 36.888	-80.551 +/- 18.074	-12.426 +/- 2.265	-122.400 +/- 57.450	75.519 +/- 63.780
4	Fel d 1-MYR	-248.775 +/- 23.314	-120.364 +/- 16.376	-18.563 +/- 0.858	-103.832 +/- 46.822	283.870 +/- 57.340
5	Fel d 1-AND	-69.937 +/- 16.538	-89.704 +/- 16.340	-10.993 +/- 1.313	-4.751 +/- 9.681	35.511 +/- 14.212
6	Fel d 1-PRO	-124.483 +/- 10.464	-164.774 +/- 9.134	-18.545 +/- 0.674	-9.532 +/- 5.512	68.368 +/- 11.292
7	Fel d 1-PRE	-61.160 +/- 9.291	-88.693 +/- 11.315	-11.393 +/- 1.189	-6.716 +/- 7.403	45.642 +/- 12.547

Figure S1. Graphical representation of Root Mean Square Deviation (RMSD) of Fel d 1 complex with 7 selected ligands.

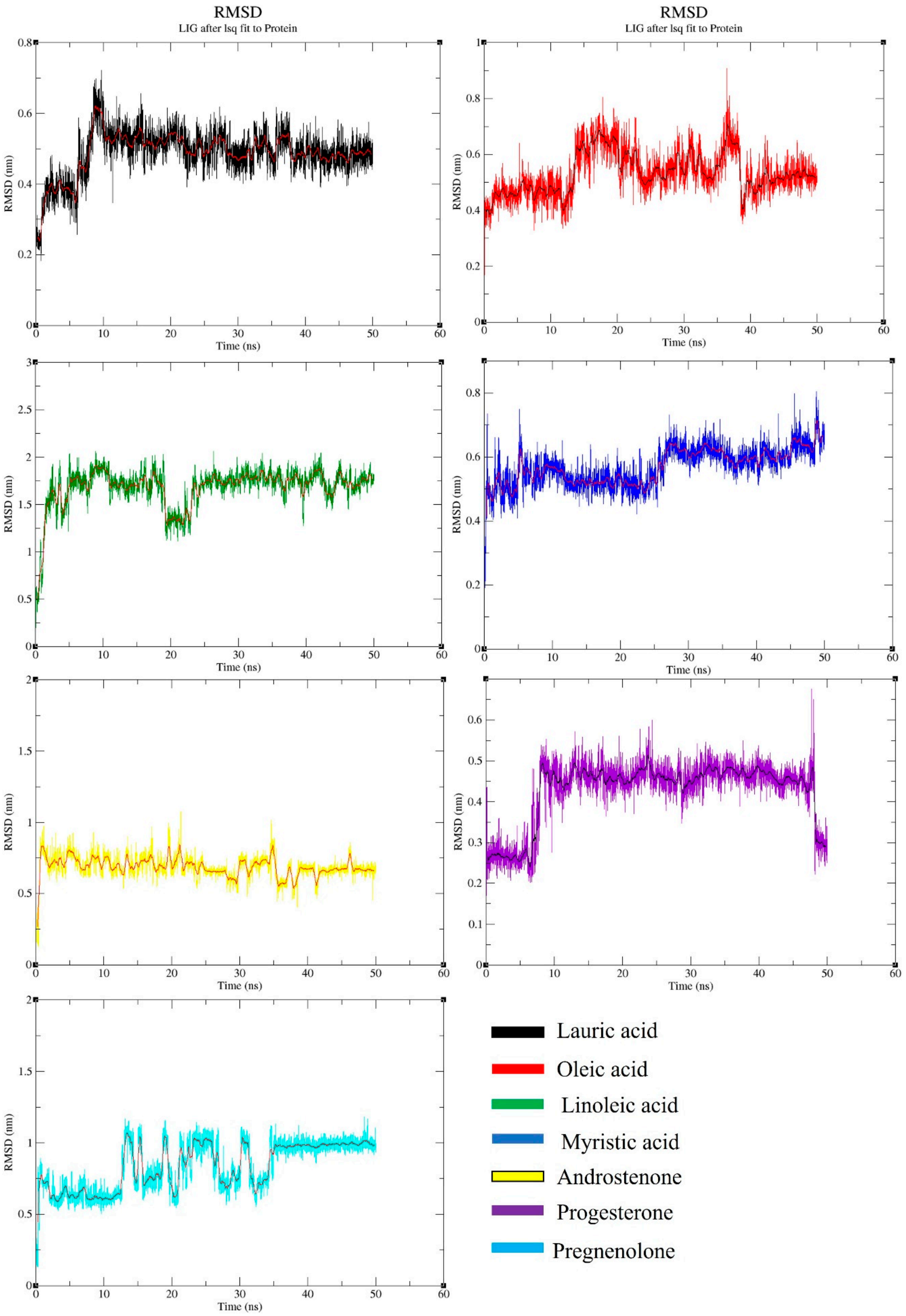

Figure S2. Combined view of H-bond interactions between Fel d 1 and 7 selected ligands.

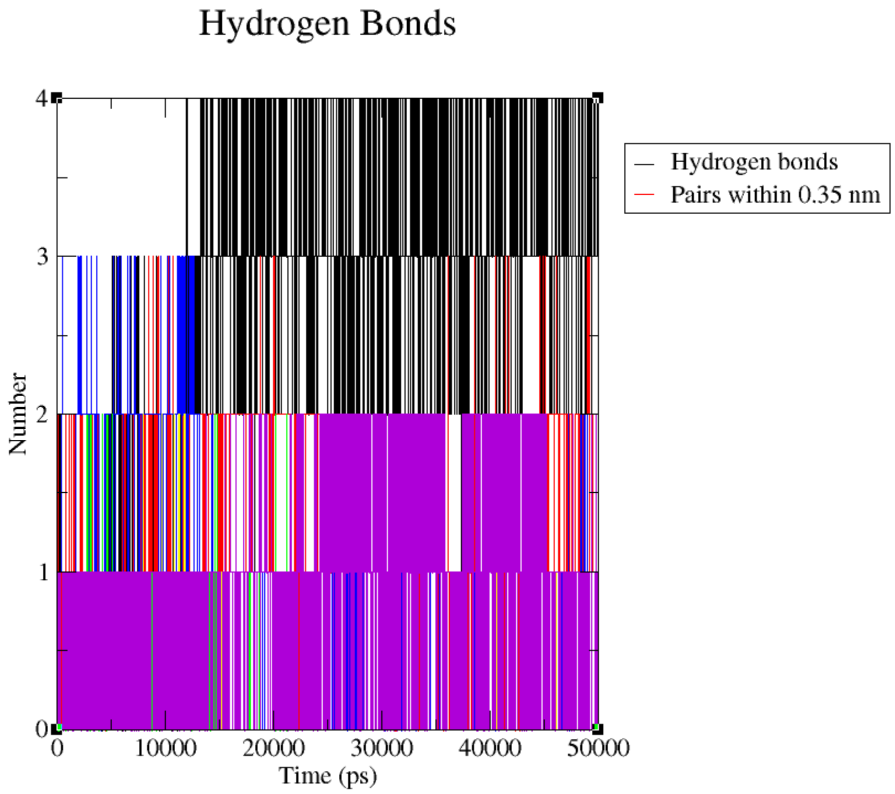

Figure S3. Surface accessible residual site in Fel d 1. The accessible residue map of full-length sequence of 2EJN dimer (A), chain 1 (B) and chain 2 (C) was displayed. The red colour line indicates the threshold point in the scale. The highly accessible residues were shown in yellow colour regions.

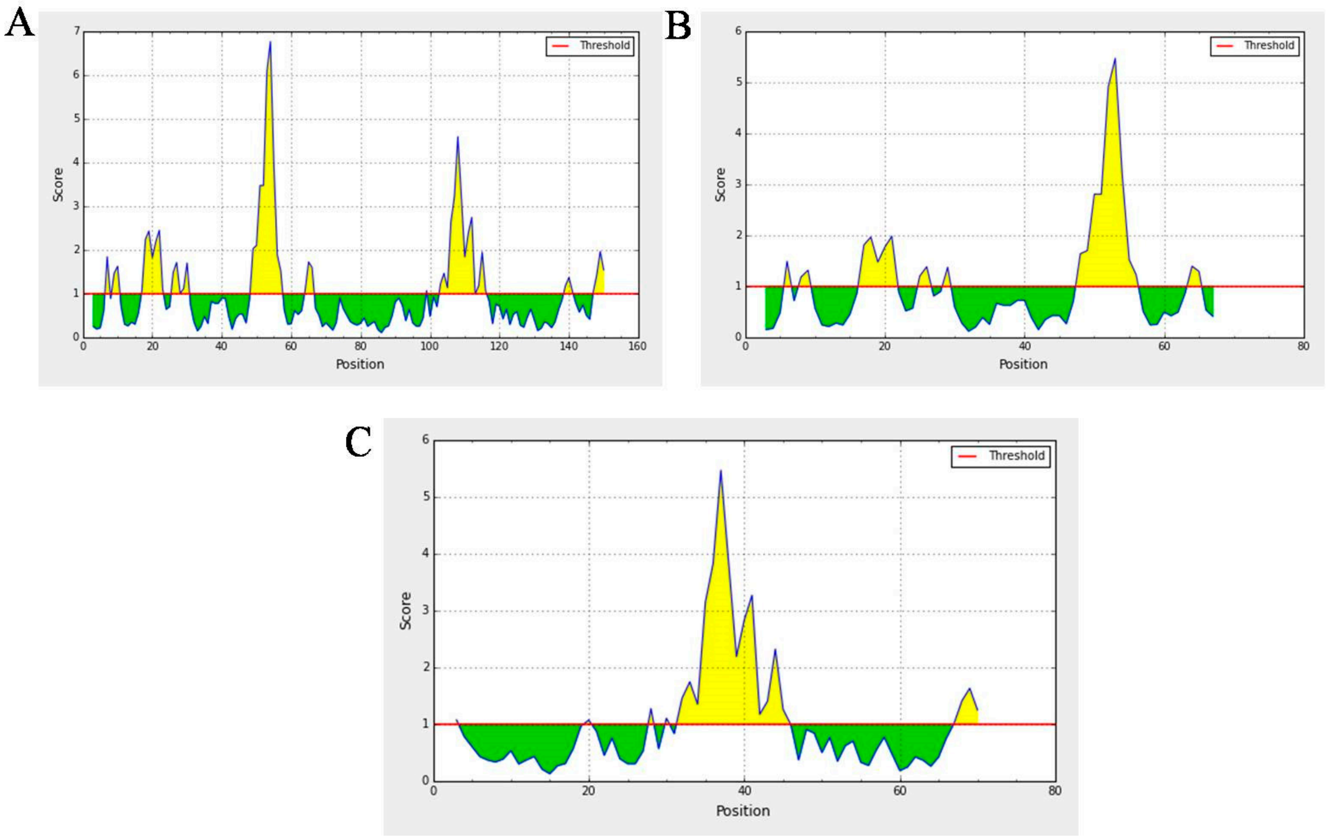

Figure S4. The antigenic determinant site was predicted in Fel d 1 using Kolaskar & Tongaonkar method from IEDB webserver. Antigenic peptide map of the dimer (A), chain 1 (B) and chain 2 (C) was displayed. The red colour line indicates the threshold point in the scale. The highest antigenic determinant site was shown in yellow colour regions.

A

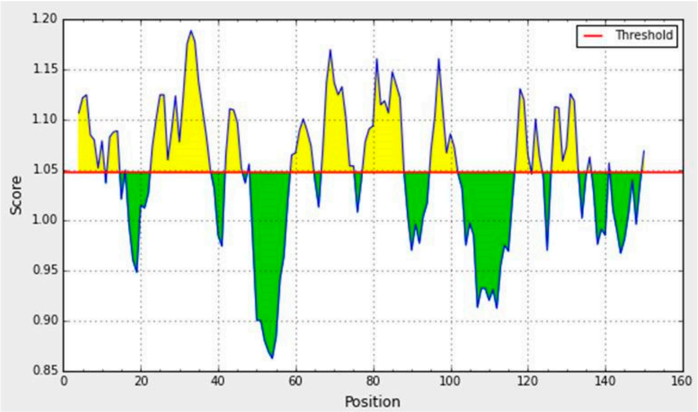

B

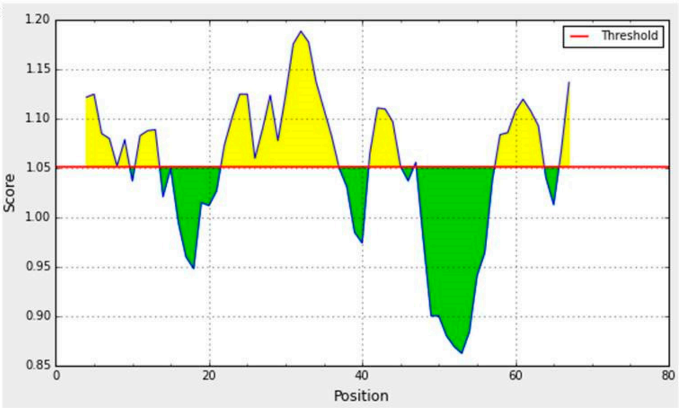

C

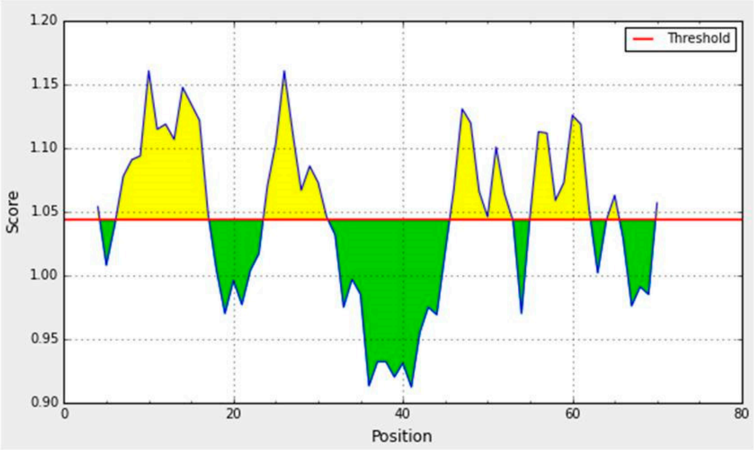

Figure S5. The predicted B-cell conformational epitope site using BEPro. The graphical plot showed epitope conservation in chain 1 (A) and chain 2 of Fel d 1 (B). The BEPro result showed that the epitopes sites showed different epitope pattern, in both chains.

A

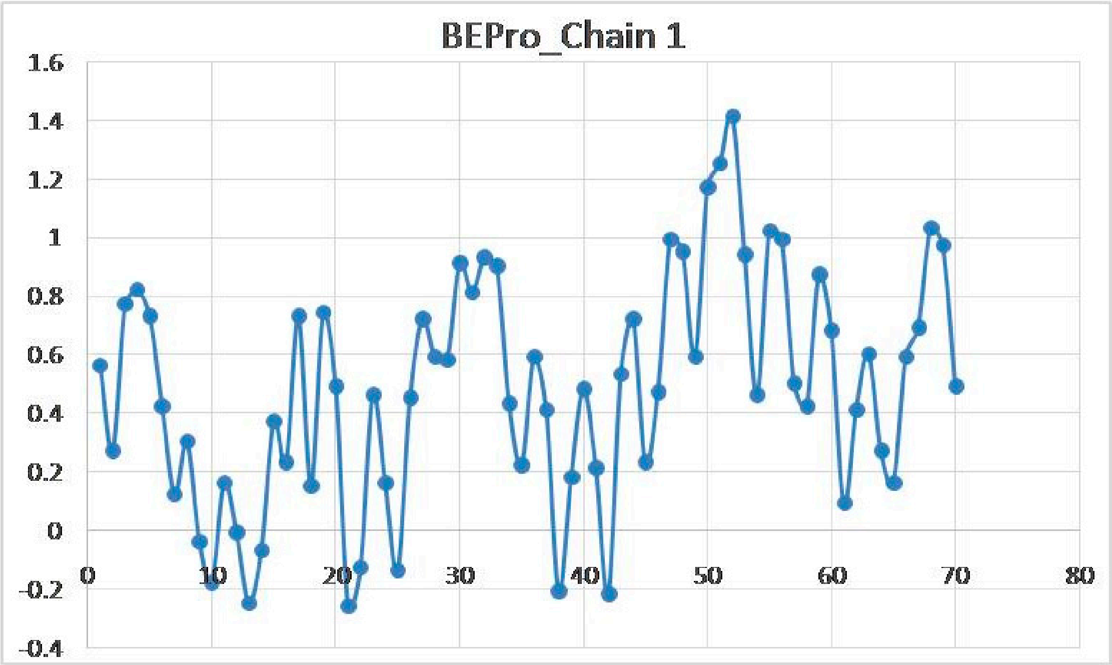

B

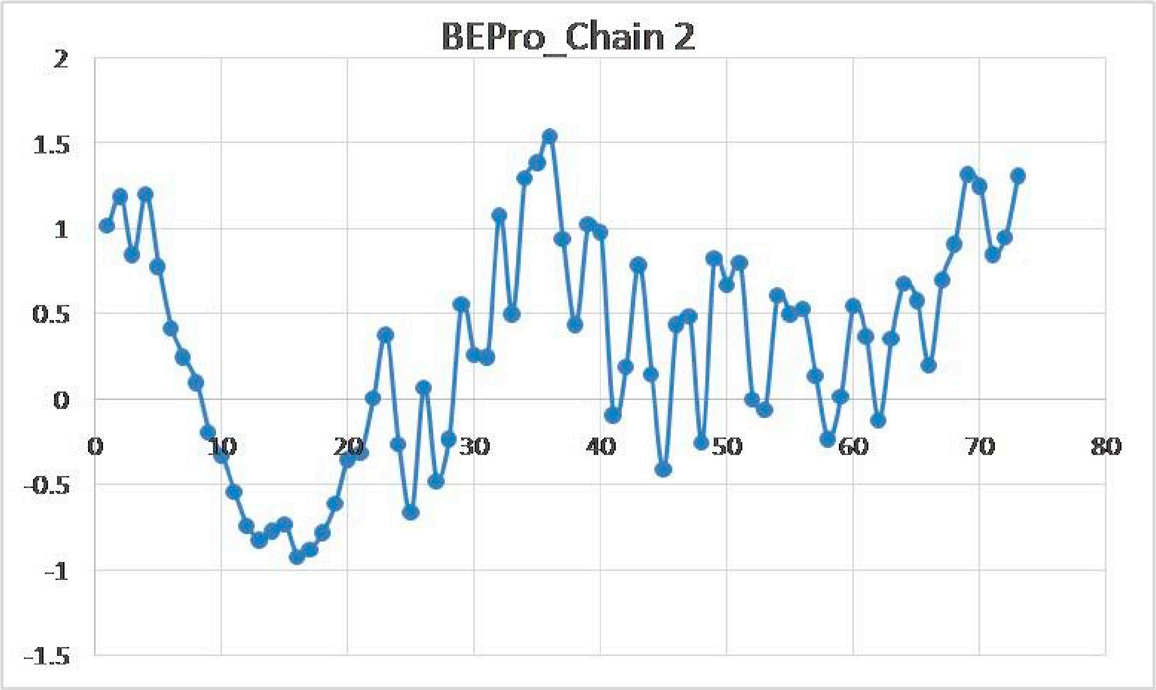

Figure S6. MDS analysis of mAb (heavy chain) bound Fel d 1 subunit A with LAU ligand. (A) The graphical representation of RMSD, (B) RMSF graph, and hydrogen bond plot (inside).

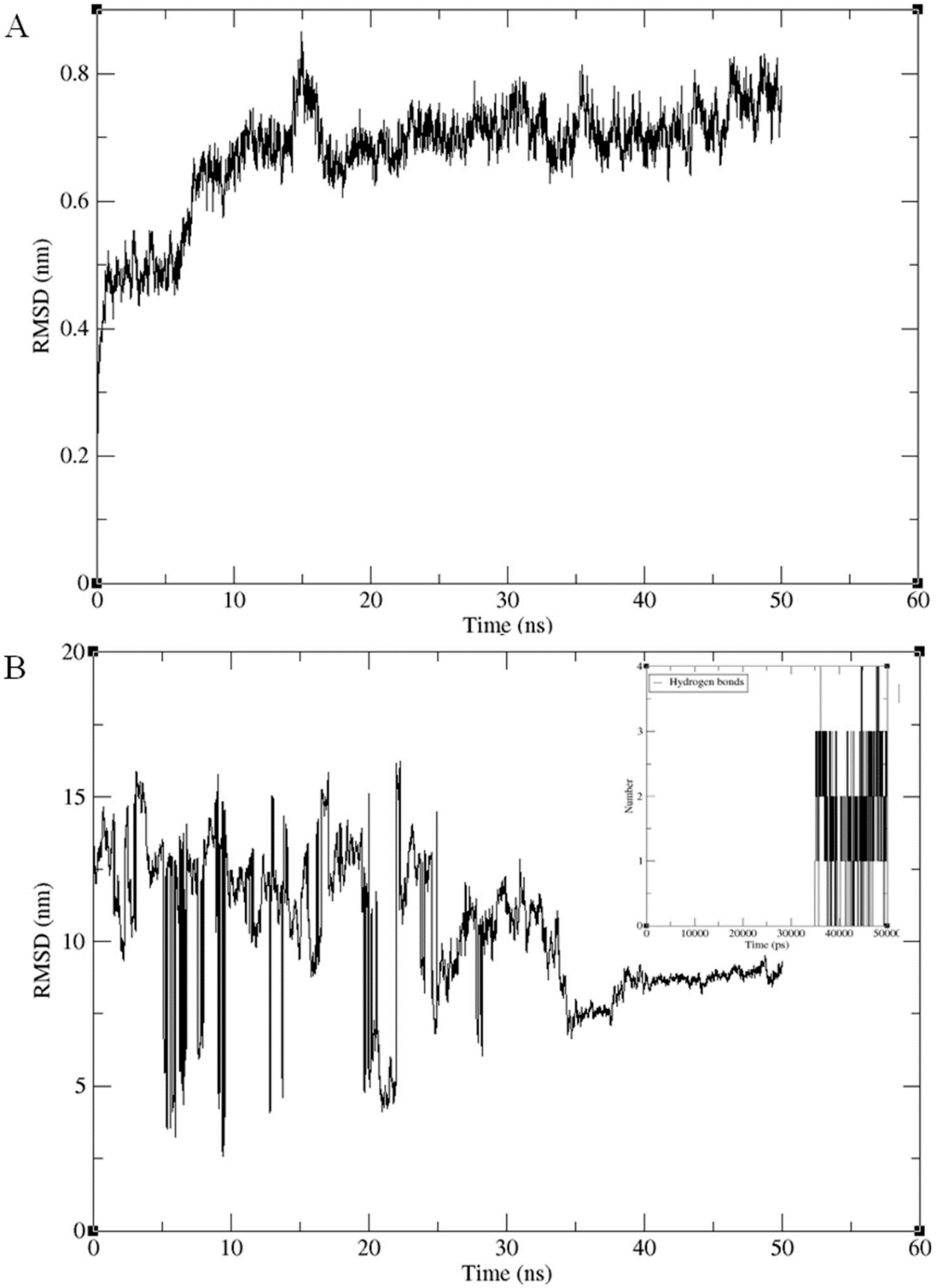

Figure S7. Sequence alignment of chains of Fel d 1 with ABP chains. Sequence similarity of chains of Fel d 1 with (A) ABPA27, (B) ABPBG27, (C) ABPBG26. The sequence alignment of Fel d 1-ABP chains contains as many identical and semi-identical residues (*) and (:) respectively. (D) Prediction of functionally exposed and structurally buried conservation sites in chain 1 and 2 of Fel d 1 with ABP subunits (ABPA-alpha; ABPBG-beta/gamma). The bold type residues of Fel d 1 were highly mapped to ABP subunits.

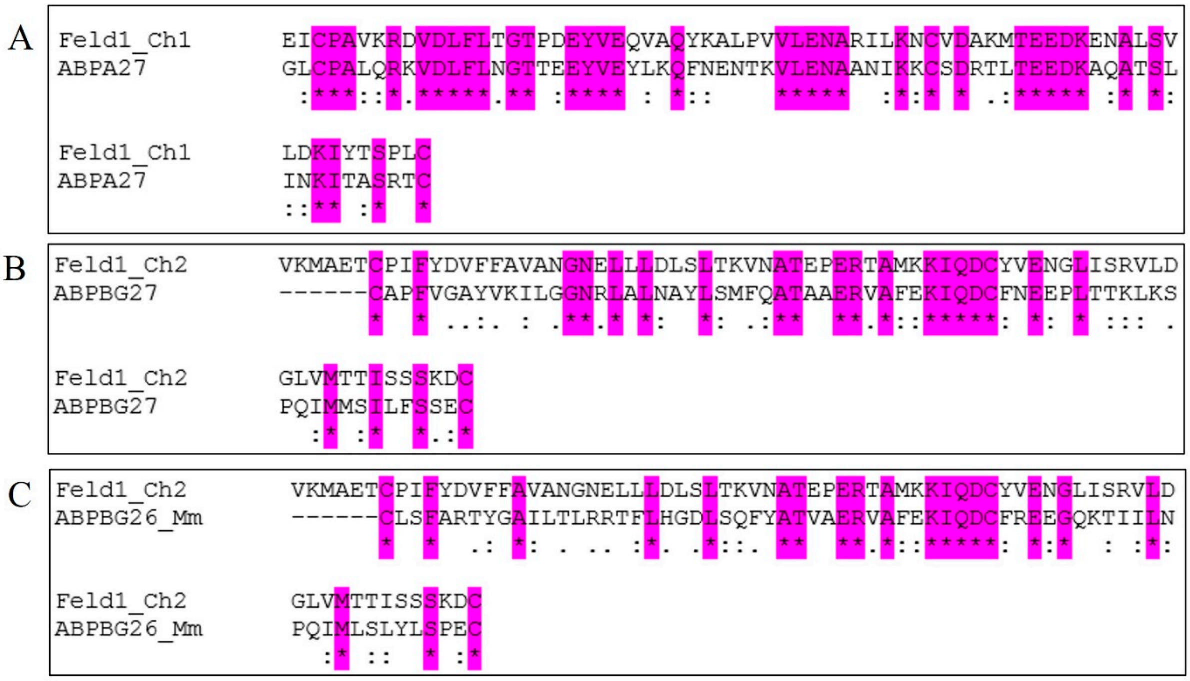

D

Conservation sites in chain 1 of Fel d 1		Conservation sites in ABPA		Conservation sites in chain 2 of Fel d 1		Conservation sites in ABPBG	
Functionally Exposed	Structurally buried	Functionally exposed	Structurally buried	Functionally exposed	Structurally buried	Functionally exposed	Structurally buried
**Cys3**	**Tyr21**	**Gly1**	**Tyr21**	**Thr29**	Phe4	Cys1	**Cys42**
**Asn37**	**Ala38**	**Cys3**	**Ala38**	**Glu32**	Leu19	Phe4	
**Lys42**		**Ala5**	Cys44	**Lys38**	Leu23	**Thr29**	
**Asp46**		**Asn37**	Leu49	**Gln40**	Ala35	**Glu32**	
**Thr50**		**Lys42**		**Glu45**	**Cys42**	Glu37	
**Glu52**		**Asp46**		**Arg51**	Cys67	**Lys38**	
**Asp53**		**Thr50**		**Ser64**		**Gln40**	
**Lys54**		**Asp53**		**Arg86**		**Glu45**	
**Lys63**		**Lys54**				**Ser64**	
**Cys70**		**Lys63**				Glu66	
